# Supplementary material for: Osteoarthritis Changes Hip Geometry and Biomechanics Regardless of Bone Mineral Density—A Quantitative Computed Tomography Study
Source: J Clin Med. 2019 May 12;8(5):669. doi: 10.3390/jcm8050669 (PMC6572464; doi:10.3390/jcm8050669)
Supplement: Supplementary file 1 [file jcm-08-00669-s001.pdf]

Table 1. Patients' baseline characteristics. Mean values of selected morphometric and densitometric characteristics in BMD-based groups.

	NORMAL (21/175)	OSTEOPENIA (72/175)	OSTEOPOROSIS (82/175)	NORMAL vs. OSTEOPOROSIS
AGE (years)	63.1	67.1	71.8	<0.00001[a]
HEIGHT (cm)	159.9	161.5	158.8	0.0017[a]
WEIGHT (kg)	64.7	69.1	60.7	<0.00001[a]
HIP OA (N)	9	38	32	0.2522[a]
NECK_ANGLE (dg)	41.8	39.7	40.6	0.07[1]
NECK_WIDTH (mm)	32.8	33.1	31.7	0.01[1]
HIP_AXIS_LENGTH (mm)	110.2	110.6	109.6	0.42[1]
TH BMD (g/cm3)	0.772	0.718	0.568	0.000000[1]
TH Volume (cm3)	266.1	244.5	198.7	0.86[1]
THC BMD (mg/cm3)	22.7	21.8	17.03	0.000000[1]
THC Volume (cm3)	29.3	30.3	29.8	0.000000[2]
THT BMD (mg/cm3)	85.6	89.7	85.6	0.05[1]
THT Volume (cm3)	0.477	0.430	0.316	0.0004[1]
FN BMD (mg/cm3)	881.8	869.8	990.9	0.000000[2]
FN Volume (cm3)	13.9	13.1	9.5	0.44[1]
TR BMD (mg/cm3)	29.3	30.3	29.8	0.04[1]
TR Volume (cm3)	16.4	15.1	9.9	0.86[1]
TRC BMD (g/cm3)	0.295	0.287	0.251	0.00001[1]
TRC Volume (cm3)	127.2	117.9	99.6	0.000000[1]
TRT BMD (mg/cm3)	8.71	8.71	7.53	0.000000[1]
TRT Volume (cm3)	29.3	30.3	29.8	0.02[1]
IT BMD (mg/cm3)	79.2	74.6	75.7	0.000000[2]
IT Volume (cm3)	0.700	0.638	0.497	0.17[1]
ITC BMD (mg/cm3)	270.5	247.3	203.6	0.00002[1]
ITC Volume (cm3)	3.3	3.1	2.3	0.000000[1]
ITT BMD (mg/cm3)	4.6	4.78	4.68	0.03[2]
ITT Volume (cm3)	12.1	12.4	11.7	0.0001[2]
WT BMD (g/cm3)	0.606	0.559	0.440	0.000000[1]
WT Volume (cm3)	205.1	184.4	147.1	0.07[1]
Troch Width (mm)	6.05	5.83	4.44	0.14[1]
Troch Height (mm)	9.9	10.5	10.1	0.19[1]

TH – total hip, THC – total hip cortical, THT – total hip trabecular, FN – femoral neck, TR – greater trochanter, TRC – greater trochanter cortical, TRT – greater trochanter trabecular, IT – intertrochanteric region, ITC – intertrochanteric region cortical, ITT – intertrochanteric region trabecular, WT – Ward's triangle. [a] - Kruskal – Wallis test (comparing all groups), [1] U Mann-Whitney test, [2] Student t-test.

Table 2. Results (p-values) of U Mann-Whitney test comparing different densitometric groups with regard to presence of radiographic signs of hip osteoarthritis.

	NORMAL	OSTEOPENIA	OSTEOPOROSIS
NECK_ANGLE	0.888	0.186	0.238
NECK_WIDTH	0.246	0.503	0.942
HIP_AXIS_LENGTH	0.081	0.0006	0.279
TH BMD	0.737	0.795	0.011
TH Volume	0.005	0.021	0.079
THC BMD	0.837	0.184	0.018
THC Volume	0.288	0.031	0.00001
THT BMD	0.228	0.499	0.232
THT Volume	0.022	0.026	0.241
FN BMD	0.106	0.307	0.004
FN Volume	0.152	0.012	0.024
TR BMD	0.610	0.334	0.054
TR Volume	0.002	0.006	0.024
TRC BMD	0.137	0.239	0.265
TRC Volume	0.461	0.806	0.019
TRT BMD	0.189	0.916	0.431
TRT Volume	0.005	0.005	0.107
IT BMD	0.736	0.164	0.001
IT Volume	0.019	0.356	0.141
ITC BMD	0.896	0.004	0.411

ITC Volume	0.088	0.041	0.00002
ITT BMD	0.356	0.243	0.164
ITT Volume	0.048	0.692	0.391
WT BMD	0.175	0.055	0.831
WT Volume	0.024	0.256	0.239
Troch Width	0.197	0.027	0.013
Troch Height	0.862	0.731	0.878

TH – total hip, THC – total hip cortical, THT – total hip trabecular, FN – femoral neck, TR – greater trochanter, TRC – greater trochanter cortical, TRT – greater trochanter trabecular, IT – intertrochanteric region, ITC – intertrochanteric region cortical, ITT – intertrochanteric region trabecular, WT – Ward's triangle.
